# Towards Designing Green-Inspired Nano- and Microemulsions Alongside Novel Solvatochromic Probes as an Effective Tool in Delivery Issues

**DOI:** 10.3390/ijms26189259

**Published:** 2025-09-22

**Authors:** Aleksandra Szarwaryn, Wojciech Bartkowiak, Tomasz K. Olszewski, Urszula Bazylińska

**Affiliations:** Department of Physical and Quantum Chemistry, Faculty of Chemistry, Wroclaw University of Science and Technology, Wybrzeze Wyspianskiego 27, 50-370 Wroclaw, Poland; aleksandra.szarwaryn@pwr.edu.pl (A.S.); wojciech.bartkowiak@pwr.edu.pl (W.B.); tomasz.olszewski@pwr.edu.pl (T.K.O.)

**Keywords:** solvatochromism, micropolarity, 5-dimethylamino-5′-nitro-2,2′-bithiophene, UV–Vis spectroscopy, nanoemulsion, microemulsion

## Abstract

The extensive use of submicron emulsion systems, particularly those stabilized by nonionic surfactants, with their proven effectiveness and safety profile, provides a reassuring foundation for our research. Consequently, we designed and engineered new submicron emulsion formulations stabilized with a biocompatible surfactant polyoxyethylated cocoamine, whose nonionic character is due to a high degree of polyoxyethylation. We chose oleic acid as the oil phase, a fatty acid known for its beneficial properties. This led to novel biocompatible nanoemulsions with high stability and cosurfactant-free microemulsions. The dynamic light scattering studies confirmed that both formulations have a nanometric size and low polydispersity index values. Moreover, transmission electron microscopy verified the nanodroplets’ morphological homogeneity and spherical shape. The resulting nanoplatforms can be applied to carry bioactive agents in the pharmaceutical and cosmetic fields. For this reason, we solubilized newly synthesized 5-dimethylamino-5′-nitro-2,2′-bithiophene as a model hydrophobic cargo for delivering poorly water-soluble compounds. This dye was chosen due to its strong solvatochromic behavior and suitability for micropolarity analysis via UV–Vis spectroscopy. We also present a simple method for rapid micropolarity screening to assess the type of nanodispersion via solvatochromic shift as an alternative procedure for evaluating of the oils used to fabricate nanoformulations for pharmaceutical and cosmetic purposes.

## 1. Introduction

Over recent years, there has been a drive to modify processes for designing stable emulsion formulations as novel and effective nanocarriers while maintaining sustainability and being environmentally friendly. Consequently, the main challenge is to maintaining high dispersion stability, while minimize potentially toxic components, preferably by replacing them with “green” alternatives. The nanoemulsions (NEs) and the microemulsions (MEs), with their extensive applications in various fields, hold promising potential as nanocarriers of bioactive substances in controlled drug delivery of cosmetics and pharmaceuticals. Regardless of the administration route, their versatility has been particularly appreciated [1,2]. Despite several compositional similarities, they differ in their physicochemical properties [3]. These distinctions are crucial for understanding their unique applications. For instance, droplet size is often insufficient as a criterion because the ranges of these two colloidal dispersions overlap. MEs are presumed to have a droplet size of 5–100 nm [4], while NEs have a size of 20–500 nm [5], although various references give slightly different ranges. Among the most crucial distinctions is the thermodynamic stability of MEs, which NEs do not possess (all the ME components are in thermodynamic equilibrium with each other). Unfortunately, to achieve this long-term stability, a very high concentration of surfactant and often an additional cosurfactant is required to develop the favorable spontaneous curvature of the interfacial layer [6]. This can increase the irritating effect of ME formulations, which is a substantial limitation for specific applications [7]. NEs, in contrast, do not need such high surfactant concentrations, and most do not involve an additional stabilizer. This type of formulation is in thermodynamic disequilibrium, which will consequently lead to the destabilization of the system. However, depending on the components involved and their compatibility with each other, this process may be prolonged. For this reason, NEs are defined as having kinetic stability, allowing them to remain very stable for a long time during storage [3]. Nevertheless, a particular challenge is providing sufficient stability to emulsions composed of polar oils that can partially mix with water, which are most endangered by destabilization processes. This is why the choice of surfactant—the main stabilizing molecule that reduces the surface tension between two initially non-mixable phases—needs to be very conscientiously considered [8]. For safety reasons and due to growing public awareness of environmental issues, there is a noticeable trend towards selecting nonionic “green” surfactants inspired by naturally occurring compounds and a significant need to design modern formulations containing biocompatible emulsifiers obtained from natural sources. Regarding the fact that the two liquid phases (oil and water) coexist in emulsion systems, from this point of view, it is also crucial to emphasize the polarity of the oil when designing the composition since the mass transfer may induce the Ostwald ripening phenomenon between dispersed droplets, ultimately destabilizing the system [9]. The solvatochromic probe can be applied as a model cargo to study the solubilization process and evaluate changes in polarity within the nanocarrier. The strong solvatochromic behavior of organic dyes could indicate a change in the microenvironment polarity, enabling the detection of even subtle differences. The dye can potentially be used to investigate whether unfavorable emulsion breakdown processes occur and to indirectly test the integrity and stability of the nanocarrier.

Since the interactions between solute and solvent are complex and often difficult to quantify, an empirical approach is often used with a suitable molecular sensor probe. The solvatochromic dyes provide a better understanding of solvent phenomena at the microscale compared to determining large-scale parameters such as the dielectric constant or refractive index. Due to this, single-parameter polarity scales based on the dye’s solvatochromic properties are effectively used as empirical indicators of the local microenvironment and have gained tremendous popularity in the past few years. This approach was also implemented as a tool for determining the polarity of the nanocarrier microenvironment [10]. Spectral changes, especially solvatochromic shifts recorded using UV–Vis spectroscopy and an appropriate molecular probe with significant solvatochromic behavior, can suggest potential solubilization sites of the bioactive substance [11]. This can help to enhance the optimization of nanoplatforms with a high loading capacity for potential use as drug delivery systems, along with providing the highest possible stability.

**Figure 1:**
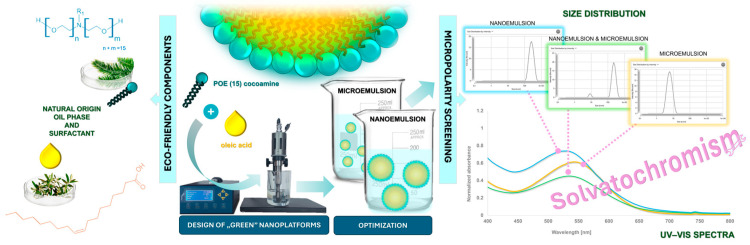
Schematic representation of the investigation into green nanoplatform design and the utilization of the solvatochromic approach.

In this manuscript, we present two biocompatible submicron emulsion dispersions: cosurfactant-free microemulsions and nanoemulsions obtained via self-assembly processes with enhanced stability. Both formulations were designed with the inspiration of green chemistry and according to sustainable development principles, alongside achieving low production costs and sustaining high stability. Their high safety might be profitably used in the cosmetic, pharmaceutical, nutriceutical, and nanomedicine fields, opening up new possibilities for product development. Moreover, we propose a novel procedure to optimize and distinguish preliminarily emulsion-type systems as well as to evaluate the polarity of oils and a newly synthesized solvatochromic probe—5-Dimethylamino-5′-nitro-2,2′-bithiophene (DNBT)— using UV–Vis spectroscopy. DNBT is a solvatochromic dye, the so-called Effenberger probe in the literature data [12]. Due to its particular importance, especially in the context of the solvatochromic phenomenon, DNBT has been studied most thoroughly both experimentally and with quantum chemical simulations [13,14,15,16,17,18,19]. The energy required to excite the molecule decreases through the more favorable interactions with polar solvent molecules, resulting in a smaller energy gap. Consequently, the excited state is better stabilized than the ground one, resulting in the bathochromic spectrum shifts (positive solvatochromism) as the solvent’s polarity increases [20]. Although this probe has been partially tested in various solvents, it has not yet been applied to assess micropolarity in more complex systems [21]. Due to its prominent properties and broad application potential, many chromophores inspired by this probe are currently being designed [22,23]. The schematic representation of the investigation into green nanoplatform design and the application of the solvatochromic approach is shown in Figure 1.

The proposed approach involves the use of solvatochromic effects for polarity profiling in the designed emulsion systems, which we believe is a significant advancement in the field. To the best of our knowledge, such a comparative analysis has not been reported before. From an application point of view, the environment evaluation inside the nanocarriers is essential. With the appropriate formulation design and selection of a suitable composition for the loaded cargo, the effectiveness and stability of their ingredients are simultaneously improved. Furthermore, the solubilization effects and micropolarity evaluations in NEs and MEs have never been compared to each other in a single study using the solvatochromic approach. There is also virtually no ongoing research regarding solvatochromism in nanoemulsions. Consequently, the choice of the solvatochromic probe is also not coincidental. It was selected from others according to the strong positive solvatochromic behavior it exhibits, along with the recognition of its lack of previous studies in more complex mixtures and microheterogenic systems. Therefore, we believe it provides a remarkable contribution to the field of supramolecular and colloidal chemistry. We also believe that the results and conclusions from this manuscript will provide a bridge to a deeper understanding of solubilization in the emulsion systems using molecular probes representing strong solvatochromic behavior.

## 2. Results and Discussion

Researchers are often inconsistent in assessing the differences between nano- and microemulsions. Fortunately, some have investigated solutions to distinguish those formulations from each other simply. Anton and Vandamme [24] propose diluting the sample, and if the oil has precipitated out, it is a microemulsion (in which case Winsor type IV will turn into Winsor type I). Nonetheless, if a minimal amount of oil is used, it can be challenging to see the oil precipitate out of the microemulsion, especially after high dilution. Similar to the effect of temperature, in the case of microemulsions, this will have a potentially significant influence on changes in their structure and, consequently, droplet size. However, in nanoemulsions, the temperature effect also affects the formulations composed of nonionic polyethoxylated surfactants, which are particularly sensitive to temperature [25]. Theoretically, leaving microemulsions and nanoemulsions under unchanged conditions for sufficiently long (often very long periods, e.g., years) will make the nanoemulsion undergo breakdown processes, while the microemulsion will remain stable [24]. However, in practice, the time-consuming nature of this approach makes it inapplicable to most research purposes, highlighting the urgent need for alternative methods to distinguish the different emulsion types.

Thakore et al. [26] also reported other ways to characterize the type of emulsion, such as mechanical stress, e.g., agitation, temperature changes (heating–cooling cycles), and then, after some time, microemulsions will return to the initial state and nanoemulsions will not. Nevertheless, it is difficult to determine how fast this process will occur in the case of microemulsions (it may take more time to return to thermodynamic equilibrium). This approach, however, is used extensively in the literature to evaluate the kinetic stability of nanoemulsions and is perfectly valid, although it may not be sufficient when trying to distinguish between these nanodispersions. Depending on the amounts of individual formulation components used, their structure (mainly the surfactant), and sometimes the ratio between oil and surfactant (and often an additional cosurfactant), microemulsion architecture can take many shapes—from simple spherical structures, called swollen micelles, to more elongated structures, bicontinuous arrangements, and even more complex ones such as hexagonal systems. Nanoemulsions, on the other hand, exist in two types—o/w (oil-in-water) and w/o (water-in-oil) dispersions—and most often have a spherical shape. Consequently, it is possible to distinguish them from each other using methods that show their droplet morphology, such as scattering (neutron, X-ray, or light) or electron microscopy methods [26]. However, this may be problematic in the case of very small droplet sizes. An appealing way to distinguish between micro- and nanoemulsion systems is to analyze the particle size distribution. If only microemulsions are present in the system, a single narrow peak will be visible, while in the case of nanoemulsions, it may be a single narrow peak, a single broad peak, or multiple peaks [3,26]. Nevertheless, this method, used as a stand-alone criterion, has limitations and cannot unambiguously confirm the emulsion type, underscoring the complexity of the topic. The examples mentioned above exemplify how challenging it is to distinguish these two dispersions from each other.

The appropriate choice of the used solvatochromic dye (DNBT) may also provide a preliminary indication of the type of formulation presented. In the case of the micro- and nanoemulsions’ coexistence, it may allow us to potentially differentiate between these systems and infer the sample’s homogeneity (if the size distribution is heterogeneous, the spectrum will be shifted).

### 2.1. Evaluation and Optimization of Pseudoternary Phase Diagrams

The pseudoternary diagrams are used to monitor phase behavior and distinguish dispersion (microemulsion, nanoemulsion, and macroemulsion) from two-phase areas and to map the specific regions of a particular formulation type. The diagrams are presented as an equilateral triangle, where each vertex indicates 100% content of a given component. While moving away from the vertex, the percentage changes, allowing for precise identification of each ingredient’s proportion required to obtain the desired system [27].

**Figure 2:**
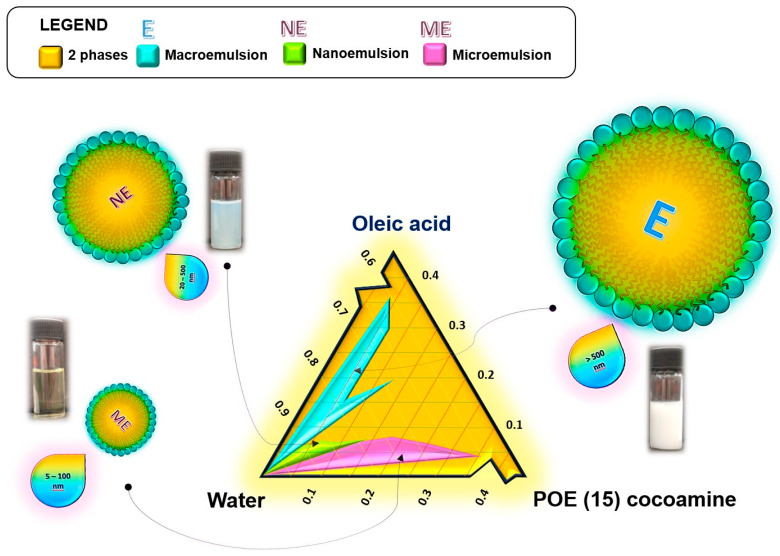
Representative partial pseudoternary phase diagram indicating the o/w macroemulsion (blue), o/w nanoemulsion (light green), o/w microemulsion (pink), and double phase (orange) regions at room temperature.

Following this, it is possible to quickly identify a ratio between the surfactant and oil phases that will be optimal for the application of particular interest. Not all oil phases are equally compatible with a given surfactant. Depending on the structure of the oil and the surfactant, there may be unfavorable interactions with the oil phase; consequently, these can lead to a significant reduction in the area appearance, characteristic of a given emulsion type in the phase diagram [28,29]. Thus, the choice of either emulsifier or oil phase is essential. This issue will be discussed in more detail in the subsequent sections of this manuscript and in the Supplementary Materials. For greater precision, only the partial pseudoternary phase diagram for oleic acid and polyoxyethylene (15) cocoamine is shown in Figure 2. Using the phase diagram, sample compositions were selected and optimized to achieve satisfactory droplet size, charge, and low polydispersity until highly homogeneous systems were obtained. Since it was well-established that microemulsion preparation generally requires high surfactant concentrations [30], the ME region dominated when the ratio of surfactant to oil phase was increased. Finally, the eight most promising ME and NE formulations were selected for further physicochemical evaluation and solubilization studies.

### 2.2. Influence of the Composition on the Formulation Stability

One of the most critical requirements for the applicability of any formulation is the maintenance of its stability over time. A significant change in the droplet size and ζ-potential during long-time storage can indicate unsatisfactory stability. For this reason, the influence of time on the formulation behavior is an essential parameter to evaluate its potential for subsequent applications. Generally, to evaluate the colloidal stability, samples are stored under controlled conditions for a certain period, and the physicochemical parameters are monitored again [31]. For this purpose, the D_H_, PdI, and ζ-potential values were measured for the fresh emulsion and after one month of its preparation.

**Figure 3:**
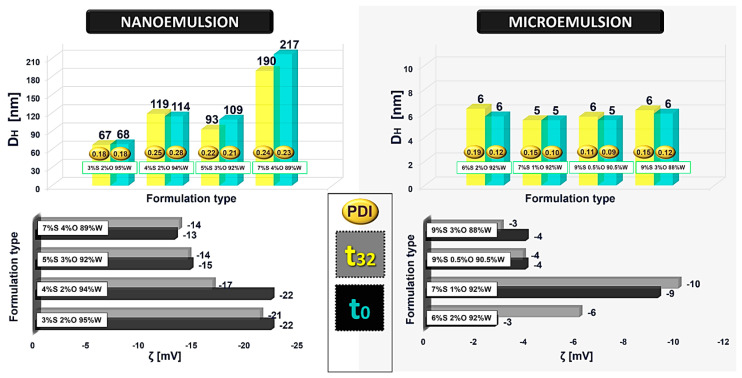
Changes in hydrodynamic diameter (D_H_) over time and changes in zeta potential for the investigated micro- and nanoemulsion systems from the time of their formation (t_0_) and after one month (t_32_).

Differences in stability between the respective formulations were investigated. The stability process was evaluated by leaving the samples in a dark place at 25 °C. The behavior of micro- and nanoemulsion systems after preparation (t_0_) and over one month (t_32_) left at room temperature is shown in Figure 3. The PdI is a parameter related to the monodispersity of the formulation. In technological applications and industrial scale-up during production, it is highly relevant that the formulation will be homogeneous as this affects its customizability properties. As shown in Figure 3, all the presented compositions are characterized by high homogeneity and uniform size, having a narrow size distribution (PdI < 0.3). Moreover, the microemulsions are characterized by an ultra-low polydispersity index (for example, in the case of the 9%S 0.5%O 90.5%W formulation, the PdI is 0.09) so there should be no issue when scaling up during processing, and highly homogeneous nanocarriers will be achieved [32].

Modifying the ratio of the surfactant to the oil phase makes it possible to adjust the formulation, consequently obtaining the desired parameter from the application point of view, e.g., the appropriate size. There is also a discernible trend that as the proportion of the oil phase to surfactant increases, the size of the droplet increases too. In the case of nanoemulsions, the smallest droplet size is characterized by dispersions with lower oil in proportion to the surfactant ratio. This correlation is less prominent in the case of microemulsions since all obtained formulations are characterized by relatively small droplet sizes (D_H_ = 5–6 nm). After one month, we observe a slight change in their droplet size. This is distinctly more pronounced for nano- than microemulsions, which is consistent with their stability profile. The difference in droplet size for the freshly prepared formulation and those stored for over one month is more tremendous for higher oil content. The formulation stability behavior is mainly related to the surfactant-to-oil ratio. The higher the percentage of oil in the formulation, the greater the tendency to destabilisation processes (coalescence, flocculation, sedimentation, or creaming) [1]. Thus, in the case of nanoemulsions with a composition of 7%S 4%O 89%W and 5%S 3%O 92%W, more remarkable changes in the size are observed. Nanoemulsions containing 2% oil phase practically do not change their size after one month. Similarly, microemulsions preserve their droplet size regardless of time since they are characterized by thermodynamic stability [3].

On the other hand, no significant changes in the ζ-potential during the formulation storage are observed; therefore, it can be considered that the colloidal stability is potentially not affected since monitoring charge changes on the nanocarrier surface is also a key parameter for determining its stability [1]. It is assumed that a high or low ζ-potential value indicates satisfactory electrostatic repulsion, which contributes to preserving the long-term stability of the nanocarrier over time. A well-established rule in the literature is that the ζ-potential should be greater than +30 or −30 mV to ensure adequate system stability. However, the chemical nature of surfactant also has a non-negligible effect on the charge [7]. POE (15) cocoamine has an interesting structure as quaternary amines are generally characteristic of cationic surfactants; however, as the number of polyoxyethylene units increases, the surfactant takes on a more nonionic character (POE (15) cocoamine has 15 polyoxyethylated units). Hence (according to the product datasheet provided by PCC Chemax manufacturing company), the cationic character of the head group is masked, and the surfactant adopts a more nonionic character, as evidenced by the ζ-potential values. Moreover, low ζ-potential values are characteristic of many nonionic surfactants, which exhibit high stability over time [7,33]. Figure 3 shows the changes in ζ-potential values over time for selected nano- and microemulsion formulations. In most cases, the ζ-potential values are practically unchanged, which confirms the hypothesis regarding the maintenance of colloidal stability of the formulations over time. Slight changes in ζ-potential can be observed only for nanoemulsions with a composition of 4%S 2%O 94%W (+5 mV) and for microemulsions with a composition of 6%S 2%O 92%W (−3 mV). Interestingly, in the case of microemulsion, the ζ-potential values deepen slightly over time, probably indicating a further stabilization of the systems. The raw DLS correlograms, along with the corresponding cumulant fits and a detailed technical note on data analysis, are presented in the Supplementary Materials.

### 2.3. Physicochemical Characteristics of Micro- and Nanoemulsion Systems with the Solubilized DNBT Probe

The bioactive agents of low water solubility generally do not show long-term stability after entering the body. Hence, various types of nanocarriers are applied to improve the bioactives’ solubility and avoid their possible degradation, protecting them from negative environmental impact. The solubilization of the hydrophobic cargo in a nanocarrier also enhances its bioavailability, which is crucial from the perspective of versatile use in nutriceutical, cosmetic, pharmaceutical, and even biomedicine applications [34]. 

Emulsion systems—as more advanced micellar formulations with distinctive structures—are well-suited for the solubilization of hydrophobic molecules due to the addition of oil, which usually localizes in the surfactant-surrounded core of the nanocarrier. Thus, it is assumed that the cargo solubilization occurs particularly in the micellar aggregate core. However, the oil molecules can adopt different positions in this surfactant aggregate, thereby influencing the nanocarrier’s interior environment (see the Section 2.4.2). By choosing the appropriate procedure of the system formation and modifying certain process parameters, it is possible to obtain the desired formulation properties, e.g., the appropriate droplet size [35].

**Figure 4:**
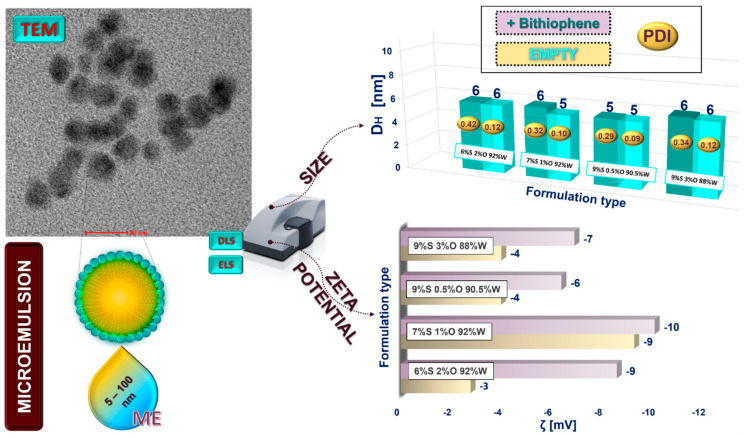
Hydrodynamic diameter (D_H_) and zeta potential values for the investigated microemulsion systems for empty nanocarriers and with cargo (bithiophene). Image made with a transmission electron microscope (TEM) showing the morphology and high uniformity of microemulsion droplets (empty 9%S 0.5% O 90.5%W formulation). Scale bar represents 20 nm.

**Figure 5:**
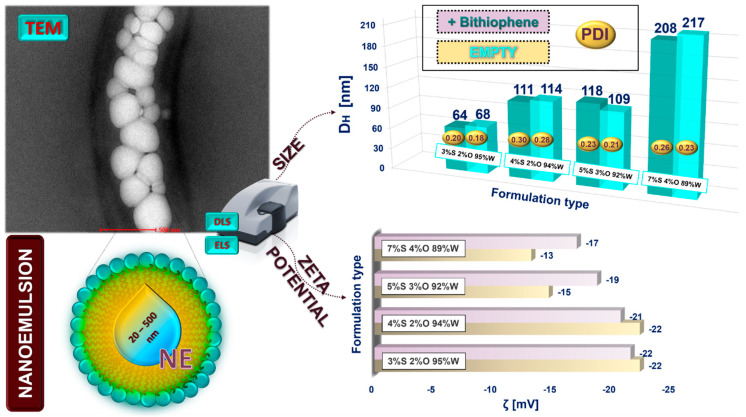
Hydrodynamic diameter (D_H_) and zeta potential values for the investigated nanoemulsion systems for empty nanocarriers and with cargo (bithiophene). Image made with a transmission electron microscope (TEM) showing the morphology and high uniformity of nanoemulsion droplets (empty 7%S 4% O 89%W formulation). Scale bar represents 500 nm.

The loaded nano- and microemulsions were prepared following the ultrasonically supported solubilization of the solvatochromic probe (DNBT) in the formed nanocarriers (the detailed compositions are shown in Figure 4 and Figure 5). The ζ-potential and hydrodynamic diameter were then measured to verify whether the DNBT molecule was solubilized inside the nanocarrier or whether it would be found on its surface [36]. Since there were no significant changes in the ζ-potential values and the droplet size increased slightly, the hypothesis of the solubilization of DNBT inside the nanoplatform could be confirmed [37]. Consequently, only slight changes in the ζ-potential values of the obtained formulations indicate the dye’s effective incorporation inside the nanocarrier. Relatively small sizes of the droplets may be connected with the method employed (the ultrasonication approach) [38]. Furthermore, both formulations show an increase in polydispersity, probably due to the placement of the dye in the nanocarrier, which is slightly more pronounced for micro- than nanoemulsions. No relevant changes in size are apparent for either emulsion type; only for the nanoemulsion formulation with 7%S 4%O 89%W is the increase in size more noticeable, which may be due to the inclusion of more oil in this formulation. The droplet size could also be influenced by the hydrophilic–lipophilic balance (HLB) value of the surfactant applied. Surfactants with higher HLB values are generally presumed to form smaller nanoemulsion droplets [35]. Small droplet sizes can be particularly beneficial, not only for transdermal applications but also for facilitated transport through capillaries. Mazonde et al. pointed out that nanocarriers with sizes of 100–150 nm cannot leave the capillaries, while smaller ones with sizes of 20 to 100 nm can successfully enter through so-called fenestrated capillaries [39]. The microemulsions with a high percentage of water in the formulation can promote increased *Stratum corneum* hydration levels, thereby improving active ingredients’ transepidermal absorption [40]. The presented systems have physicochemical properties (size, PdI, and ζ-potential values) suitable for biomedical, pharmaceutical, and cosmetic applications. On this basis, it can be concluded that, after DNBT solubilization (as a model solute), the presented micro- and nanoemulsion formulations successfully prove themselves for other applications such as nanocarriers of biologically active agents, which can be incorporated inside the nanoplatform, enhancing their stability.

Since the transmission electron microscope (TEM) is a widely used technique for imaging the morphology of nanoemulsions [41], this method provided precise visualization of the morphology and stability of the obtained formulations. The nanoemulsion appears as densely packed structures, suggesting a high concentration of the relatively monodisperse droplets in the sample but without the presence of coalescence, which is validation for colloidal stability. It confirms the spherical shape and high uniformity of the o/w nanoemulsion droplets (see Figure 5 and the Supplementary Materials, Figure S1A,B). The size obtained previously from the DLS measurements, as well as the monodisperse size distribution (similar dimensions of the droplets), was also confirmed by this technique. The surfactant assured stability to external stresses as the o/w nanoemulsions preserved their spherical shape despite the sample being dried out before measurement. The black smudge underneath the nanoemulsion droplets is probably the oil droplets to which the nanocarriers have attached. It was visualized due to the different electron densities between them. The TEM technique is not the most suitable for visualizing the morphology of microemulsions because, during the preparation, water must be evaporated when the sample is dried, which can sometimes cause deformation and artifacts (caused by changes in temperature and concentration).

The selection of an appropriate surfactant and its optimal ratio compared to the other components of the composition are crucial to maintaining the stability and morphology of the system. The chosen proportions of the formulation indicate that POE (15) cocoamine is very effective in the stabilization of micro- and nanoemulsion systems because, despite the drying of the samples, the images show a classic morphology for this type of structure. Due to the relatively small size of microemulsions (5–6 nm), the resolution of TEM images was affected. The TEM images (see Figure 4, Figure S2A,B) show microemulsions with a spherical shape and a high degree of homogeneity (only small agglomerations were visible, see Figure S2B). Compared to the nanoemulsions, the contrast of the microemulsion droplets was usually lower as a result of their lower electron density. These results confirmed the relatively small size of the microemulsion formulations obtained by DLS. The microemulsions in the TEM images are slightly larger than those obtained by DLS, probably because of the drying process. The favorable physicochemical properties of the nano- and microemulsions, allowing stability over time and sustaining homogeneity in terms of size and morphology, predestine the compositions for further applications; hence, the optimized samples were subjected to consecutive analyses.

### 2.4. Monitoring Local Micropolarity

#### 2.4.1. Comparison of the Model Dye UV–Vis Spectra in Pure Solvents

The classic approach for solvatochromism studies is the comparative method, in which solutions of a solvatochromic probe are prepared in several pure solvents characterized by different polarities, and then UV–Vis spectra are measured. The polarity of the probe’s microenvironment can be assessed, indirectly demonstrating its preferential solvation in more complex mixtures such as binary or microheterogeneous systems [42].

**Figure 6:**
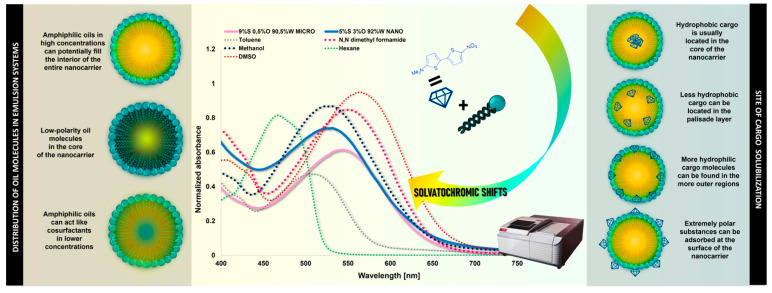
UV–Vis spectra for investigation of the solvatochromic behavior of bithiophene in pure solvents, as well as nanoemulsion and microemulsion systems. The potential site of cargo solubilization can be inferred from solvatochromic shifts observed with a micropolarity-sensitive probe.

Consequently, the UV–Vis spectra of DNBT in different solvents are shown in Figure 6, and the wavelength maxima are presented in Table S1 in the Supplementary Materials. Hexane was used as the reference nonpolar solvent, while DMSO was the most polar one. It should be noticed that DNBT has very limited solubility in water. It exhibits strong solvatochromic behavior, observable as extensive bathochromic shifts by λ_max_ = +92 nm between hexane and DMSO (467 → 559 nm). The solvatochromic behavior of DNBT was analyzed in detail many times in other studies [12,13,14,15,16,17,18,19]. Here, it should be recalled that the strong positive solvatochromic shift of the DNBT low-lying absorption band maximum as a function of solvent polarity may be mainly connected with the significant change in their dipole moment accompanying electronic excitation (π-π*) from ground to excited state (µ_g_ < µ_e_). The considered low-lying excited state has a strong charge-transfer (CT) character.

From the point of view of our investigation, the preliminary assessments of local micropolarity—based on the spectra of DNBT in pure solvents—have practical implications. The results of such analyses can be extrapolated to other microenvironments, providing a method to estimate the micropolarity inside specific types of nanocarriers indirectly. This awareness can guide the design and evaluation of the proposed formulations as potential nanocarriers in various applications.

#### 2.4.2. Assessing Local Micropolarity Inside Various Formulations

Considering the characteristic structure of o/w emulsion systems, the hydrophobic cargo is expected to be inside the core of the surfactant aggregate. However, depending on the chemical nature of the solute, the structure of the surfactant, and the oil’s nature, there are several alternative potential locations for the loaded bioactive agent. It can be found in the core, palisade layer, or even in the more outer regions, and sometimes extremely polar substances can be adsorbed at the surface [43]. Figure 6 schematically shows the possible solubilization sites in different one-phase (solvents) or three-phase systems (nano- and microemulsions). These aforementioned parameters influence the microenvironment prevailing inside the formed nanocarrier and can significantly affect the micropolarity of the interior and, thus, the solubilization efficiency. For the bioactive ingredient to maintain its therapeutic efficacy and adequate stability (critical in controlled drug delivery), the environment inside the nanocarrier is crucial. The internal conditions of the nanoplatform can also be influenced by the preparation method, the type of nanocarrier, and its composition.

Micro- and nanoemulsions can potentially co-exist in a system composed of the same surfactant, oil, and water but with a different surfactant–oil ratio (SOR) and at the appropriate temperature state condition. In the case of microemulsion formation, it is essential to maintain the proper ratios of all system components to achieve thermodynamic equilibrium between them. It can be allowed by enough free energy in the system to promote the occurrence of the most stable emulsion type. However, not all surfactants will be able to form microemulsions, and often, the addition of a cosurfactant will be required. For this reason, more surfactants can form nanoemulsions than microemulsions [26]. In our studies, a model compound, DNBT, was dissolved in more advanced micellar-type nanocarriers (micro- and nanoemulsions), and then the UV–Vis spectra were measured. Moreover, microemulsions were obtained without an additional stabilizer (without cosurfactant); thus, the UV–Vis spectra for both emulsion formulations can be compared. Significant solvatochromic shifts were observed, potentially suggesting a different polarity environment between the interiors of these systems. To more accurately evaluate solubilization patterns, the formulations were also compared with pure solvents (Figure 6). Solvatochromic behavior studies were also performed on pure oils commonly used in pharmaceutical and cosmetic compositions. A table with the wavelength maxima values (Table S1) and the corresponding UV–Vis spectra (Figure S3) are available in the Supplementary Materials.

**Figure 7:**
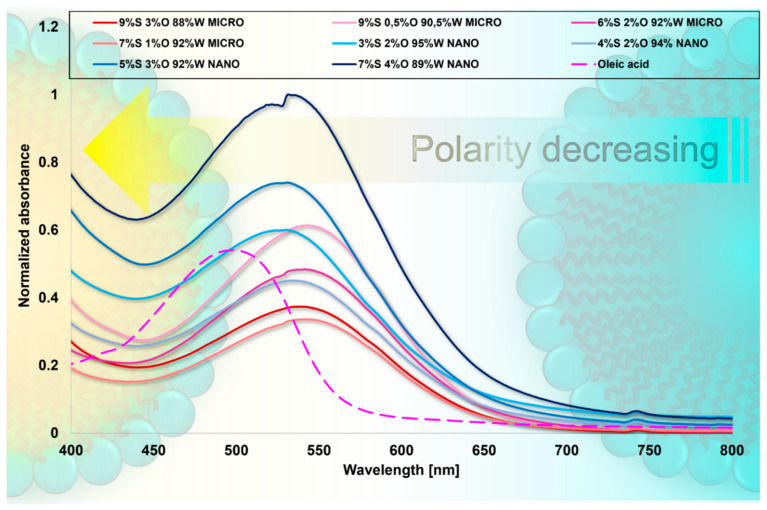
UV–Vis spectra for investigation of the solvatochromic behavior of bithiophene in nanoemulsion and microemulsion systems in comparison with bithiophene in the pure oil phase (oleic acid). Solvatochromic shifts suggest potential changes in the polarity of the local microenvironment inside different types of nanocarriers.

Shifts in the UV–Vis spectra between the different micellar types were noted, as shown in Figure 7. The maxima of the wavelengths are presented in Table S1 in the Supplementary Materials. At the same time, a tendency to localize at wavelengths characteristic of a specific formulation type is evident (all nanoemulsions localize at similar wavelengths, as do microemulsions). Nanoemulsions were most shifted toward shorter wavelengths (λ_max_ = +63 to +67 nm relative to hexane), probably as a result of their highest oil phase content. The environment inside the nanoemulsions mimicked the polarity prevailing in solvents as methanol (λ_max_ = 530 nm), acetone (λ_max_ = 531 nm), chloroform (λ_max_ = 533 nm), and dichloromethane (λ_max_ = 535 nm). Remarkably, it is not the percentage of oil phase in the formulation that is important but the ratio between the amount of surfactant and oil. In microemulsion formulations, more surfactant is needed relative to oil than in nanoemulsions; however, at the same time, in the case of the 4%S 2%O 94%W composition, the size distribution showed two subpopulations in terms of size—in addition to the peak at the nanoemulsion, a smaller peak in terms of the droplet size appeared (shown on Figure 1) and this was also noticed by a bathochromic shift. While comparing the 3%S 2%O 95%W and 4%S 2%O 94%W formulations, it can be perceived that increasing the amount of surfactant and leaving the oil concentration constant can cause a red shift by λ_max_ = +4 nm (530 → 534 nm). The absorption spectra of both 3%S 2%O 95%W and 7%S 4%O 89%W (both are nanoemulsion formulations) localize at approximately the same wavelength (530 nm and 531 nm, respectively). In the case of microemulsions, leaving the surfactant content at the same level but reducing the oil concentration of the formulation can substantially affect shifts toward longer wavelengths. Considering the formulations 9%S 3%O 88%W and 9%S 0.5%O 90.5%W, a noticeable bathochromic shift by λ_max_ = +6 nm (538 → 544 nm) can be perceived. On the other hand, the polarity is approximate to N,N-dimethyl formamide (λ_max_ = 549 nm). This may indicate a slightly different microenvironment inside these nanocarriers. The use of oleic acid seems to play a significant role as the oil molecules may localize not in the core of the nanocarrier but penetrate further into the palisade layer, depending on the oil’s concentration and character (amphiphilic) [44]. This penetration potentially leads to a more polar microenvironment inside the nanocarrier, and it is mainly observable in microemulsion formulations, probably due to the lower concentration of the oil phase and the smaller size of these nanoplatforms [45]. This indicates the sensitivity of this method to detect even the most subtle changes in micellar systems. The obtained results are as reasonable as can be expected given the physicochemical differences between the two emulsion types.

The process parameters (homogenization time, cycle, amplitude, etc.) and formulation composition (type of surfactant, oil phase, and the ratio between components) can be readjusted to achieve the most desirable physicochemical characteristics for the target application of interest. Employing solvatochromic dyes to monitor the internal environment can also help optimize drug delivery systems for various applications. This approach can be applied in several ways—such as to monitor the microenvironment inside a variety of nanocarriers (for the evaluation of whether the interior of the nanoplatform is a suitable environment for solubilization of the chosen bioactive agent depending on its hydrophobicity); in addition, it can be used to assess the integrity and stability of nanocarriers by monitoring solvatochromic shifts (if some instability occurs, then the spectrum will be shifted towards more nonpolar solvents). Another appealing way to possibly apply a solvatochromic probe and UV–Vis spectroscopy is to potentially indirectly distinguish micro- and nanoemulsions from each other at the condition that both types of colloidal dispersions could be formed using the same surfactant and oil phase. That is due to solvatochromic probes being extremely sensitive to micropolarity changes [46]. Since a UV–Vis spectrophotometer is easily accessible in almost every laboratory, this approach can potentially be applied as an alternative to other meticulous ways of improving and optimizing the fabrication process.

## 3. Materials and Methods

### 3.1. Materials

The nonionic surfactant POE (15) cocoamine was purchased from PCC Chemax (Piedmont, CA, USA). The employed oil phase was oleic acid from POCH S.A. (Gliwice, Poland). Paraffin oil was obtained from Sigma Aldrich Chemical Company (Poznań, Poland), and Isostearyl palmitate, Isopropyl palmitate, Ethylhexyl palmitate, Coco caprylate/caprate, Diisopropyl sebacate were obtained from Stearinerie Dubois Fils (Ciron, France). The following solvents—DMF, DMSO, methanol, ethanol, DCM, acetone, THF, toluene—were obtained from POCH S.A. (Gliwice, Poland). Acetonitrile and hexane were provided by Sigma Aldrich (Poznań, Poland). Chloroform was purchased from Chempur (Piekary Śląskie, Poland). The water used for the experimental part was distilled and purified using a system HLP Smart Hydrolab (Labsystem S.C., Kraków, Poland).

### 3.2. Synthetic Procedure for 5-Dimethylamino-5′-Nitro-2,2′-Bithiophene (DNBT)

**Scheme 1:**
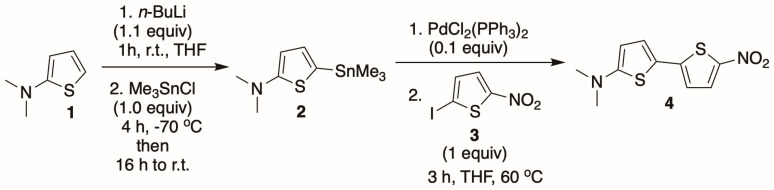
Synthetic pathway leading to 5–dimethylamino-5′–nitro-substituted 2,2′–bithiophene (DNBT) 4.

The DNBT dye (**4**) was synthesized in a two-step reaction sequence according to the modified procedure (Scheme 1) [47]. In the first stage, the 2-(dimethylamino) thiophene **1** was lithiated with *n*-BuLi at room temperature, followed by transmetalation with trimethyltin chloride at −70 °C to give the organotin compound **2**. The latter was used in the second reaction step and coupled at 60 °C with the 2-iodo-5-nitrothiophene **3** under PdCl_2_ (PPh_3_)_2_ catalysis to yield the desired bithiophene **4** with 67% yield as dark red-purplish solid. The structure of the DNBT dye (**4**) was fully confirmed by standard spectroscopic techniques, and the obtained data were in full agreement with the previously reported literature data [47].^1^H NMR δ_H_ (300 MHz, CDCl_3_): 3.01 (s, 6H), 5.81 (d, *J* = 4.2 Hz, 1H), 6.73 (d, *J* = 4.4 Hz, 1H), 7.14 (d, 1 *J* = 4.3 Hz, 1H), 7.76 (d, *J* = 4.5 Hz, 1H). Mp (°C) 176–178 (lit. [47] 177–178). The related ^1^H NMR δ_H_ (300 MHz, CDCl_3_) spectra are included in the Supplementary Materials (Figure S4).

### 3.3. Construction of Ternary Phase Diagrams

For examination, the phase behavior of the system consisted of polyoxyethylene (15) cocoamine used as a surfactant (S), oleic acid applied as an oil phase (O), and distilled water (W), a pseudoternary diagram was constructed by an aqueous titration–ultrasound method. Water was added stepwise to the previously weighed surfactant–oil mixture (SOR ratios of 9:1, 8:2, 7:3, 6:4, 5:5, 4:6, 3:7, 2:8, and 1:9, respectively). The amount of aqueous phase introduced was adjusted to achieve a water concentration of 5% to 99% of the total volume at approximately 5% intervals. After each part, the samples were mixed and then placed in thermostated tubs at a constant temperature of 20 °C for 2 min to equilibrate. The phase boundaries correspond to identical distances between subsequent experimental observations on both sides of the phase boundary. Visual observations were conducted after each 5% addition of the aqueous phase to the oil–surfactant mixture and subsequent equilibration. Visual observations resulted in the following categories: (I) transparent—oil-in-water (o/w) microemulsion (ME); (II) translucent with a blue sheen—oil-in-water (o/w) nanoemulsion (NE); (III) milky or cloudy—emulsion (E); (IV) two separate phases (2Ph).

### 3.4. Solubilization of the DNBT in Nano- and Microemulsion Systems

DNBT solubilization was performed by mixing the dye of 57.14 µg/mL concentration with the surfactant and the oil phases; then, water was added, and ultrasonic homogenization was performed. All the formulation compositions (ME or NE) were selected from the phase diagram regions and mixed by an ultrasound treatment using a tip sonicator (Hielscher Ultrasonics GmbH, Teltow, Germany) with an amplitude of 100% and a cycle of 0.6. Throughout the experiment, the sample was submerged in an ice bath to facilitate ultrasound emulsification while maintaining the processing temperature of 20 °C and minimizing the thermal effect. The procedures used for preparing empty base formulations according to the phase diagram have been disclosed in patents P.449470 (Bezkosurfaktantowe mikroemulsje oraz sposób ich wytwarzania/Surfactant-free microemulsions and preparation method thereof) [48] and P.449471 (Biokompatybilna nanoemulsja typu olej w wodzie i sposób jej wytwarzania/Biocompatible oil-in-water nanoemulsion and preparation method thereof) [49]. Additional information regarding the impact of the oil phase on emulsion stability and cargo solubilization is provided in the Supplementary Materials [50,51,52].

### 3.5. Dynamic Light Scattering (DLS): Particle Size and Polydispersity Index

The measurements of the average particle size (D_H_) and polydispersity index (PdI) values of the nano- and microemulsions were determined using the dynamic light scattering (DLS) method. A Malvern Zetasizer Pro (Blue) apparatus (Malvern Instruments, Worcestershire, UK) with an angle of incidence of light equal to 173°. Z-average and polydispersity index (PdI) were reported by averaging three subsequent instrument runs, each consisting of at least twenty measurements in disposable polystyrene cuvettes, conducted at 25 °C. The samples were measured without any dilution, and additional technical details on dynamic light scattering are provided in the Supplementary Materials [53,54,55,56,57,58], including Figures S5–S10. 

### 3.6. Electrophoretic Light Scattering (ELS): Zeta Potential

The zeta (ζ) potential, was evaluated for the measurement of the nanodroplets’ charge using electrophoretic light scattering (ELS) by applying the Smoluchowski equation to the electrophoretic mobility. The ELS measurements were performed using a Malvern Zetasizer Pro (Blue) apparatus (Malvern Instruments, Worcestershire, UK). An average of three measurements, each with at least twenty runs, was provided for the findings. To verify the stability of the system, Z-Average, PdI, and ζ-potential measurements were conducted once more following an appropriate incubation period of 32 days at 25°C.

### 3.7. Shape and Morphology Determination

The nano- and microemulsion structures and morphology were imaged using transmission electron microscopy (FEI Tecnai G2 XTWIN, Hillsboro, OR, USA). Before the imaging evaluation, a few drops of the non-diluted formulations were placed on a perforated carbon-film-coated copper grid and allowed to dry for about one hour at room temperature. Some of the samples were stained with 2% uranyl acetate before shooting.

### 3.8. UV–Vis Micropolarity Assessment

The polarity in different environments was studied by measuring the UV–Vis spectra of the prepared samples containing the DNBT solubilized in micro- and nanoemulsion systems in quartz cuvettes with an optical path length of 1 cm, using a UV/Visible double-beam spectrophotometer (Halo DB-20S). The spectra of the samples were monitored over the influence of different compositions, different micellar systems (nano and microemulsions), and in comparison with pure solvents with various polarities. Considering the small size (and thus the relatively high transparency of the systems), the samples were not diluted before measurement to reduce the effect of compositional change on the properties of the systems.

## 4. Conclusions

In the presented paper, the biocompatible submicron emulsion formulations with improved stability were revealed, i.e., the nanoemulsions with a high level of homogeneity as well as microemulsions that do not require the additional use of a cosurfactant. The compositions consist of oleic acid as a biocompatible oil phase, an aqueous phase, and a polyoxyethylated cocoamine as a potentially “green” emulsifier. The optimized procedure leads to a reduced cost of obtaining the product and, simultaneously, the potential toxicity of the system. The physicochemical properties (nanometric size, narrow size distribution, high ζ-potential values, and droplet homogeneity) of the proposed formulations predestine them as primary nanoplatforms for many applications.

Moreover, we demonstrated a significant solvatochromic behavior of the newly synthesized dye (5-dimethylamino-5′-nitro-2,2′-bithiophene) to show the high solubilization efficiency of the obtained formulations and potentially offer appealing insights to infer differences between various types of micellar systems. For instance, it is also possible to indirectly evaluate the potential location of the applied hydrophobic agent and confirm that it is inside the nanoplatform. This is crucial since different bioactive substances have varying requirements for the micropolarity of the interior of the nanocarrier. By comparing the solvatochromic shift in nano- and microemulsions, it can be seen how much the components and the nature of the nanocarrier can affect the environment inside. The results suggest a potential difference in micropolarity between the studied dispersion interiors. The experiments also showed a bathochromic shift of the emulsion formulations relative to the probe spectrum of pure oleic acid. This finding may indicate that the oil molecules were potentially simultaneously in the core and may also be imprisoned between surfactant tails, changing the microenvironment to a more polar one, which was visualized as the solvatochromic shift. For this reason, it is crucial to rationally design nanoplatforms for a given application—in particular, meticulously selecting the process conditions and individual formulation components and carefully maintaining appropriate ratios between them. The environment inside the nanocarrier is very influential in the solubility of bioactive substances. Hence, precisely evaluating the micropolarity of the nanoplatform’s interior to optimally adapt it to the cargo is undoubtedly a key element.

The demonstrated procedure is also suitable as a valuable tool for fast monitoring of the polarity of ingredients applied to cosmetic and pharmaceutical formulations, e.g., oil phases. It could be an alternative to the surface tension measurement techniques, allowing rapid screening. This approach has not yet been presented in the scientific literature and can bring many advantages. It must be highlighted that the sustainable nanoplatforms presented in this paper were designed with inspiration from green chemistry. They can be a promising tool for delivering bioactive agents with prospective various industrial applications due to their physicochemical characteristics, great colloidal stability and, especially, their potentially reduced toxicity due to biocompatible natural-origin components.

## 5. Patents

P.449470: patent application, A. Szarwaryn & U. Bazylińska, *Bezkosurfaktantowe mikroemulsje oraz sposób ich wytwarzania [Surfactant-free microemulsions and preparation method thereof]*. Patent No. P.449470. Application for Granting a Patent Adopted in the Patent Office of the Republic of Poland on 7 August 2024. Unpublished yet.

P.49471: patent application, A. Szarwaryn & U. Bazylińska, *Biokompatybilna nanoemulsja typu olej w wodzie i sposób jej wytwarzania [Biocompatible oil-in-water nanoemulsion and preparation method thereof]* Application for Granting a Patent Adopted in the Patent Office of the Republic of Poland on 7 August 2024. Unpublished yet.

## Data Availability

All the data supporting the findings of this study are available within the article. Raw data are available from the corresponding authors upon reasonable request.

## Supplementary Materials

The following supporting information can be downloaded at https://www.mdpi.com/article/10.3390/ijms26189259/s1.
