# Supplementary material for: Towards Designing Green-Inspired Nano- and Microemulsions Alongside Novel Solvatochromic Probes as an Effective Tool in Delivery Issues
Source: Int J Mol Sci. 2025 Sep 22;26(18):9259. doi: 10.3390/ijms26189259 (PMC12470261; doi:10.3390/ijms26189259)
Supplement: Supplementary file 1 [file ijms-26-09259-s001.zip › ijms-3794476-supplementary.pdf]

Supplementary Materials

Towards Designing Green-Inspired Nano- and Microemulsions Alongside Novel Solvatochromic Probes as an Effective Tool in Delivery Issues

Aleksandra Szarwaryn, Wojciech Bartkowiak, Tomasz K. Olszewski and Urszula Bazylińska *

Department of Physical and Quantum Chemistry, Faculty of Chemistry, Wroclaw University of Science and Technology, Wybrzeze Wyspianskiego 27, 50-370 Wroclaw, Poland; aleksandra.szarwaryn@pwr.edu.pl (A.S.); wojciech.bartkowiak@pwr.edu.pl (W.B.); tomasz.olszewski@pwr.edu.pl (T.K.O.)

* Correspondence: urszula.bazylinska@pwr.edu.pl

Table S1. λ_{max} values and shifts of Bithiophene in various pure solvents, nanoemulsions, microemulsions, and oils.

PURE SOLVENTS			NANOEMULSIONS			MICROEMULSIONS			OILS		
**SAMPLE**	**λ_{max}**	**SHIFT (reffering to hexane)**	**SAMPLE**	**λ_{max}**	**SHIFT (reffering to hexane)**	**SAMPLE**	**λ_{max}**	**SHIFT (reffering to hexane)**	**SAMPLE**	**λ_{max}**	**SHIFT (reffering to hexane)**
Hexane	467	-	3%S 2%O 95%W	530	+63	9%S 3%O 88%W	538	+71	Paraffin oil	479	+12
Toluene	508	+41	7%S 4%O 89%W	531	+64	6%S 2%0 92%W	541	+74	Oleic acid	496	+29
Ethanol	518	+51	5%S 3%O 92%W	531	+64	7%S 1%O 92%W	541	+74	Isostearyl palmitate	498	+31
Tetrahydrofuran	520	+53	4%S 2%O 94%W	534	+67	9%S 0.5%O 90.5%W	544	+77	Isopropyl palmitate	501	+34
Methanol	530	+63							Ethylhexyl palmitate	502	+35
Acetone	531	+64							Coco caprylate/caprate	503	+36
Chloroform	533	+66							Diisopropyl sebacate	514	+47
Dichloromethane	535	+68									
N,N-dimethyl formamide	549	+82									
Dimethyl sulfoxide	559	+92									

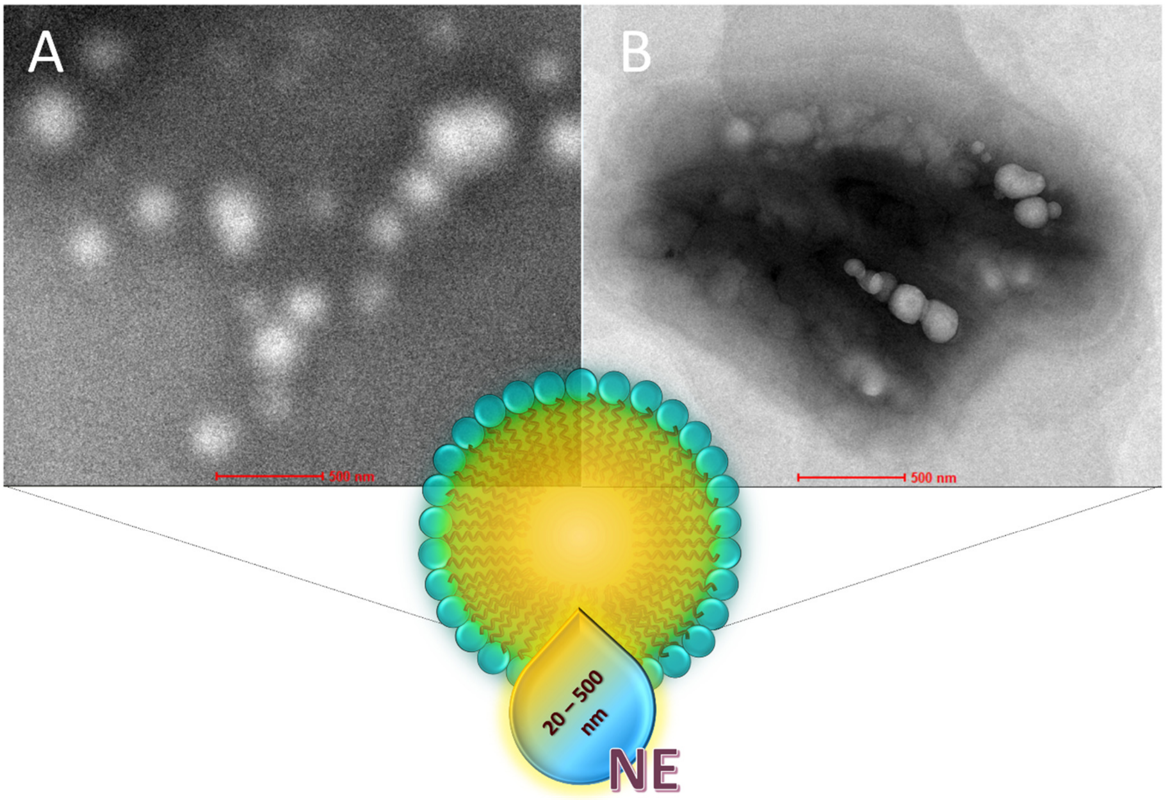

Figure S1. TEM images of the nanoemulsions (A) 5%S 3%O 92%W and (B) 4%S 2%O 94%W formulations. The scale bar represents 500 nm.

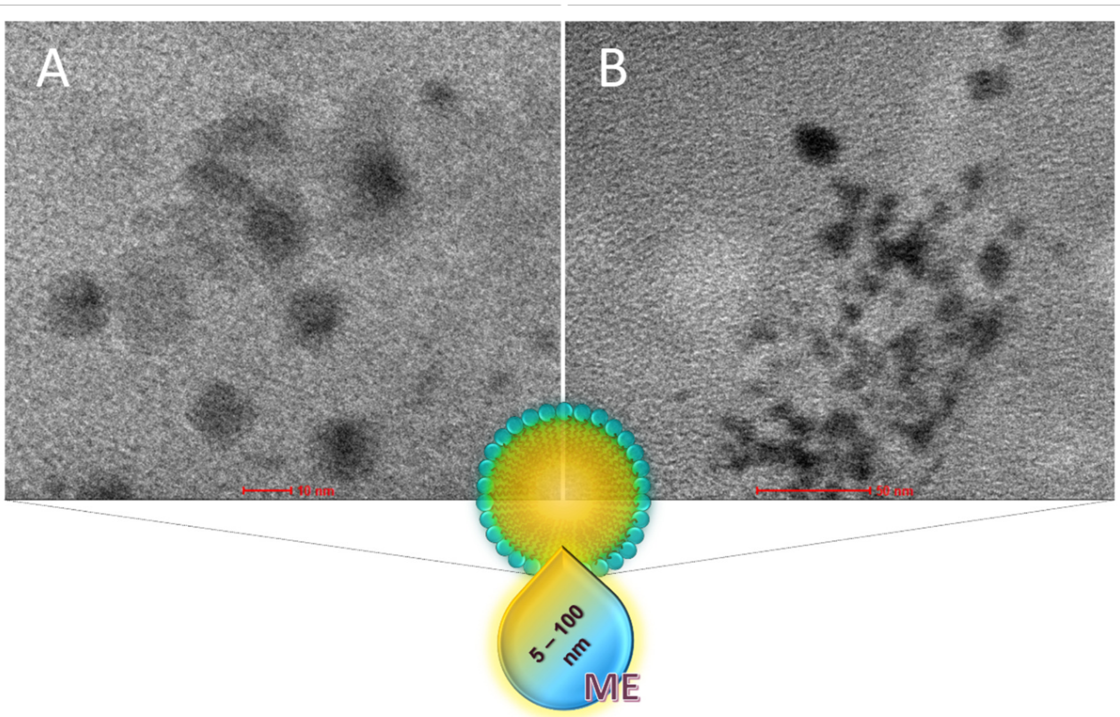

Figure S2. TEM images of the microemulsions (A) 7%S 1%O 92%W and (B) 6%S 2%O 92%W formulations. The scale bar represents (A) 10 nm and (B) 50 nm.

Impact of the oil phase for emulsion stability and cargo solubilization

Among the most important issues for selecting an oil phase is the need to ensure effective solubilization of the bioactive substance. An effective one, that is, allows for solubilization of the cargo to the desired level from a given application perspective. This is often the initial point for developing formulations of a given emulsion type for further implementation. Additionally, the properties of the oil phase itself (including whether it has any additional health-promoting benefits) are also validated, and then its compatibility with the chosen surfactant is examined. This approach markedly amplifies the solubilization efficiency and can vastly enhance the drug's therapeutic potential. The oil polarity can affect the physicochemical properties of the final emulsion formulation created, including, e.g., droplet size and stability [50].

Emollients, essential ingredients in many products, serve a dual role in cosmetic and pharmaceutical formulations. Their ability to moisturize the skin and enhance permeation is crucial for transdermal delivery [51]. Figure S3 showcases a variety of oils suitable for nano- and microemulsion compositions, selected for their high applicability in the cosmetic and pharmaceutical industries. The proposed solvatochromic probe was used to determine their polarity, and the UV–Vis spectrophotometric measurements were conducted. The spectra of the DNBT in the potential oil phases are presented in Figure S3. The wavelength maxima are summarized in Table S1.

Oils are inherently low in polarity due to the hydrocarbon chain, although classification is still evident between nonpolar mineral oils and more polar ones. The oil polarity is mainly influenced by chain length, chain branching, and the presence of additional groups such as carboxyl and ester groups [51]. The balance between all influencing factors frames the proper polarity of oil phases. This is especially relevant because oil phases are often mixtures of different compounds. The longer the chain, the more nonpolar the nature of the oil. Furthermore, the absence of additional branching and functional groups causes paraffin oil to be found at shorter

wavelengths. In the case of oleic acid, a red shift by λ_{max} = +17 nm (479 → 496 nm)—probably related to the carboxyl group—was noticed. The other esters are found at similar wavelengths with a bathochromic shift by λ_{max} = +5 nm (498 → 503 nm) from the isostearyl palmitate to coco caprylate/caprate. The presence of more than one ester group significantly increases the polarity, thus diisopropyl sebacate is shifted most bathochromically compared to other oils.

A significant bathochromic shift by λ_{max} = +35 nm (479 → 514 nm) between paraffin oil and diisopropyl sebacate has been observed, indicating a significant difference in polarity between these oils. We also performed some pilot experiments and pointed out that when diisopropyl sebacate was used as the oil phase, the microemulsion region did not appear. Most of the pseudoternary phase diagram area was a double phase or emulsion, only a tiny nanoemulsion region with large D_H (>450 nm) was observed. It is important to notice that the choice of the right surfactant and oil phase is of key importance in the context of the final product attributes.

According to the literature, the three most common methods currently used to characterize the polarity of oils include the following: oil dielectric constant, HPLC, and surface tension measurements [9]; however, these methods possess significant limitations. The first method is not always correct enough. The HPLC chromatography is often used but is susceptible to contamination. On the other hand, the surface tension measurements (considered as the gold standard) require meticulous calibration and can be affected by factors such as temperature and humidity. In contrast, the UV–Vis spectroscopy is a practical and straightforward method that can quickly determine the polarity of the oil. Hence, this technique (offering efficiency and simplicity) was applied in our investigations for quick indirect screening of the right oil phase for further emulsion formulation.

Chanama *et al.* pointed out that oil polarity significantly affects emulsion stability. Formulations with oils of lower polarity are generally more stable, while those of high polarity can also be stable when provided with a suitable surfactant, which can significantly lower the surface tension and, thus, stabilize the emulsion strongly

enough. The authors showed that regardless of the miscibility of the oil with water and its polarity, it is possible to effectively improve emulsion stability and avoid coalescence and Ostwald ripening by choosing the right stabilizer. All tested emulsions were stable using the nonionic surfactant Tween 20 as an emulsifier [8]

The length of the hydrocarbon chain, and the subsequent decrease in oil polarity, makes it more challenging to mix the oil with water. While it may seem logical to choose only nonpolar oils, it is important to note that different active substances have varying polarity requirements for their solubilization to be effective. For instance, substances that function as UV filters are better solubilized in more polar oils, such as diisopropyl sebacate [52]. This underscores the complexity of the oil selection process and the need to consider different polarity requirements.

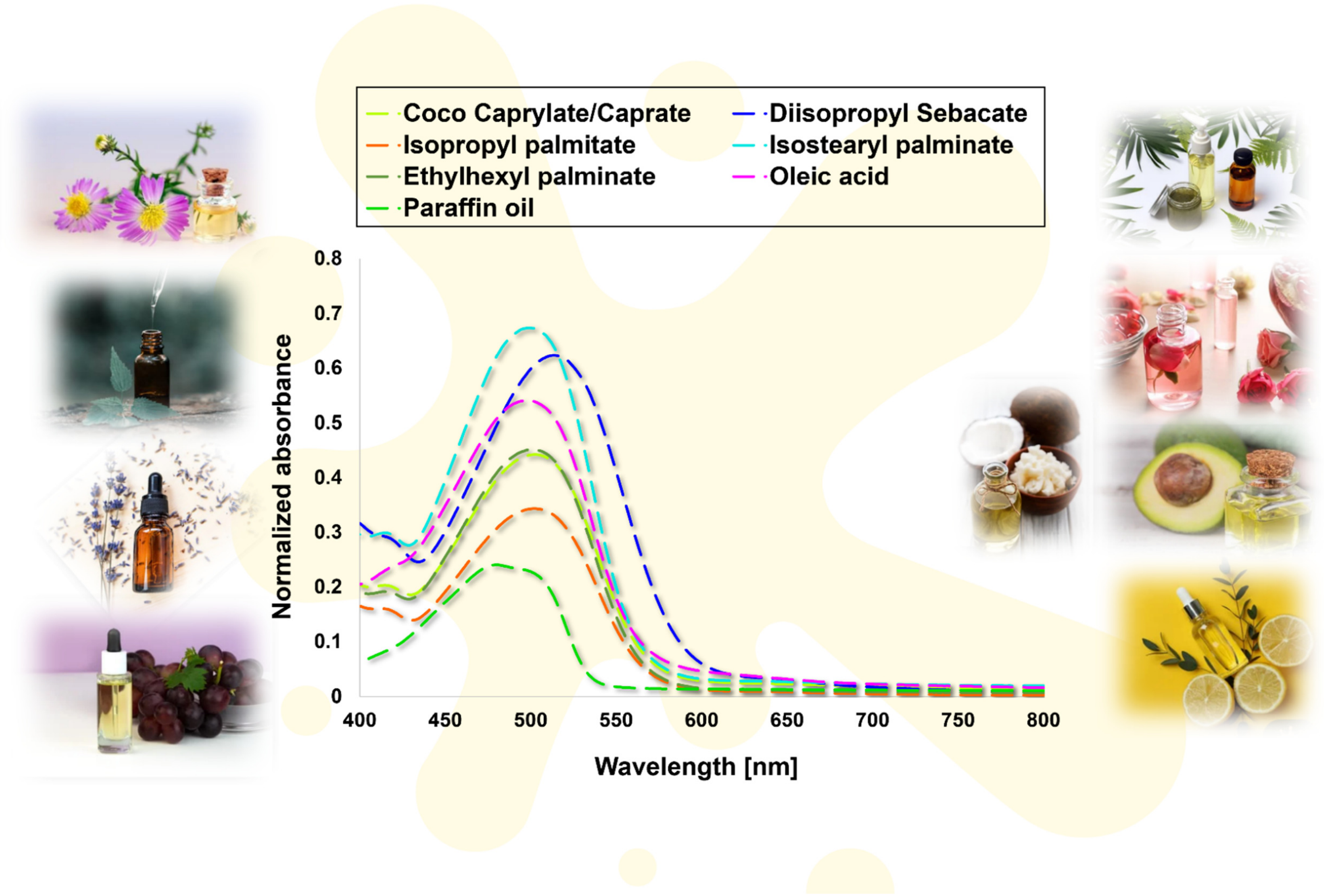

Figure S3. UV–Vis spectra for investigation of the polarity of different commonly used oil phases in the cosmetic and pharmaceutical fields.

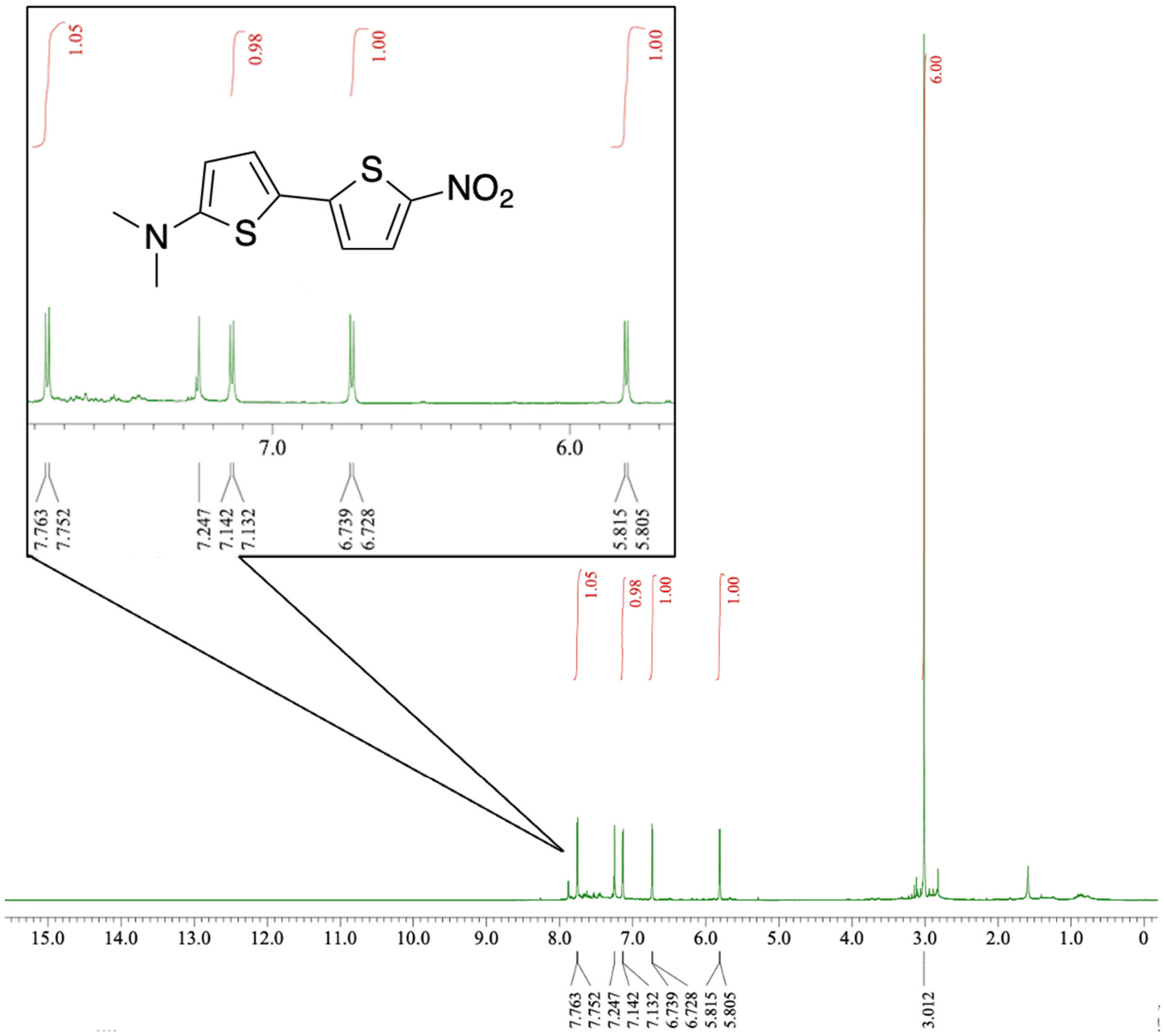

Figure S4. ^{1}H NMR (300 MHz, $CDCl_3$) spectra of the synthesized 5-dimethylamino-5′-nitro-substituted 2,2′-bithiophene (the DNBT dye).

Dynamic light scattering (DLS) - Technical note

In DLS measurements, the raw data consist of raw correlation $g_2(q, \tau)$ data to the size/radii distribution functions. These plots serve as the basis for further processing, e.g., in the calculation of particle size distributions. In order to ensure the reliability of the data, each sample was measured at least three times under identical experimental conditions. The resulting correlograms and derived particle size distributions were highly reproducible across replicates, indicating measurement stability and confirming the validity of the analytical protocol. Due to the minimal variability observed, a single representative dataset is presented. Thus, we have included representative charts recorded in the ZS Xplorer software below.

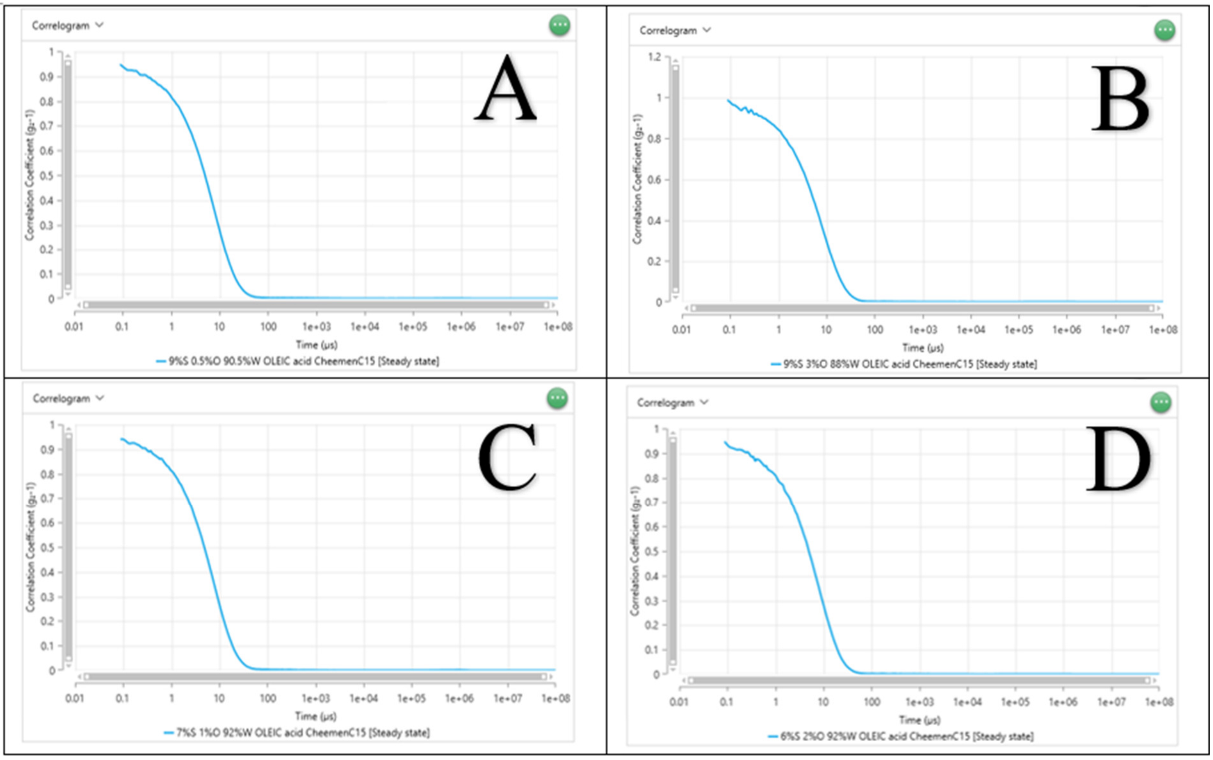

Figure S5. Autocorrelation function for the studied microemulsion systems. Formulations: **A**—9% POE(15)cocoamine / 0.5% oleic acid / 90.5% water; **B**—9% POE(15)cocoamine / 3% oleic acid / 88% water; **C**—7% POE(15)cocoamine / 1% oleic acid / 92% water; **D**—6% POE(15)cocoamine / 2% oleic acid / 92% water.

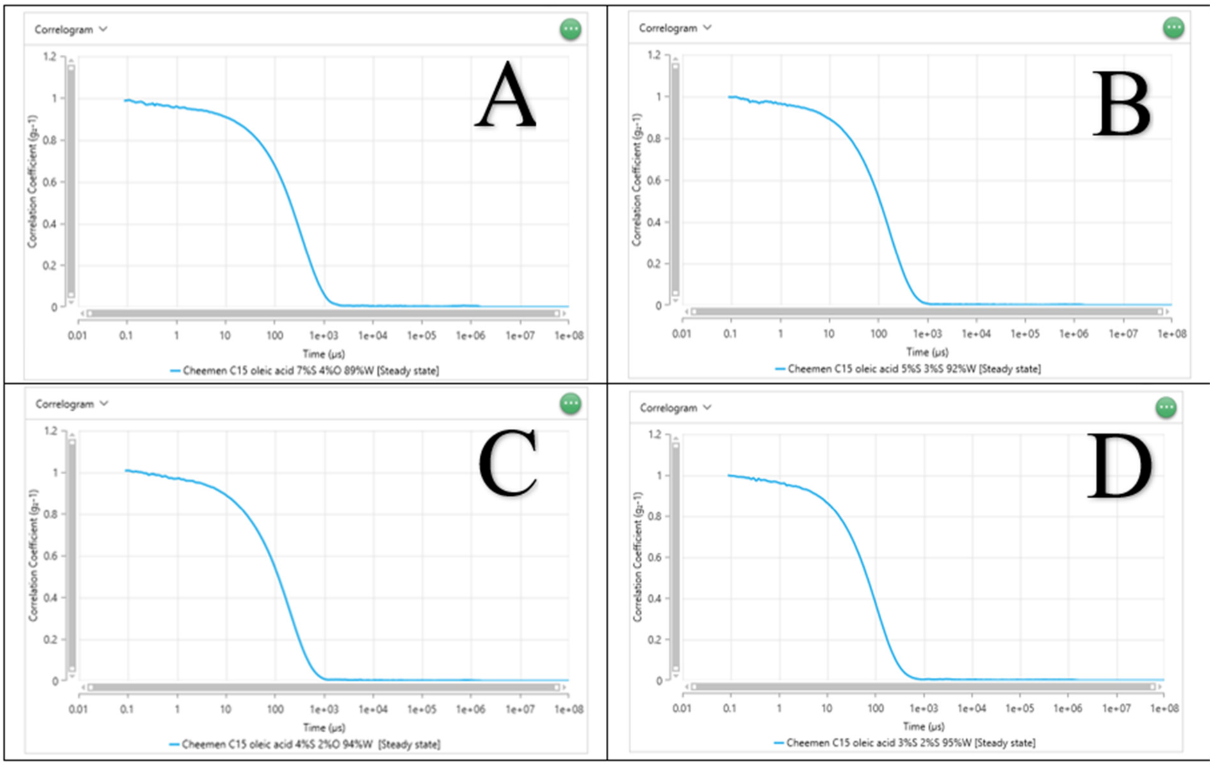

Figure S6. Autocorrelation function for the studied nanoemulsion systems. Formulations: **A**—7% POE(15)cocoamine / 4% oleic acid / 89% water; **B**—5% POE(15)cocoamine / 3% oleic acid / 92% water; **C**—4% POE(15)cocoamine / 2% oleic acid / 94% water; **D**—3% POE(15)cocoamine / 2% oleic acid / 95% water.

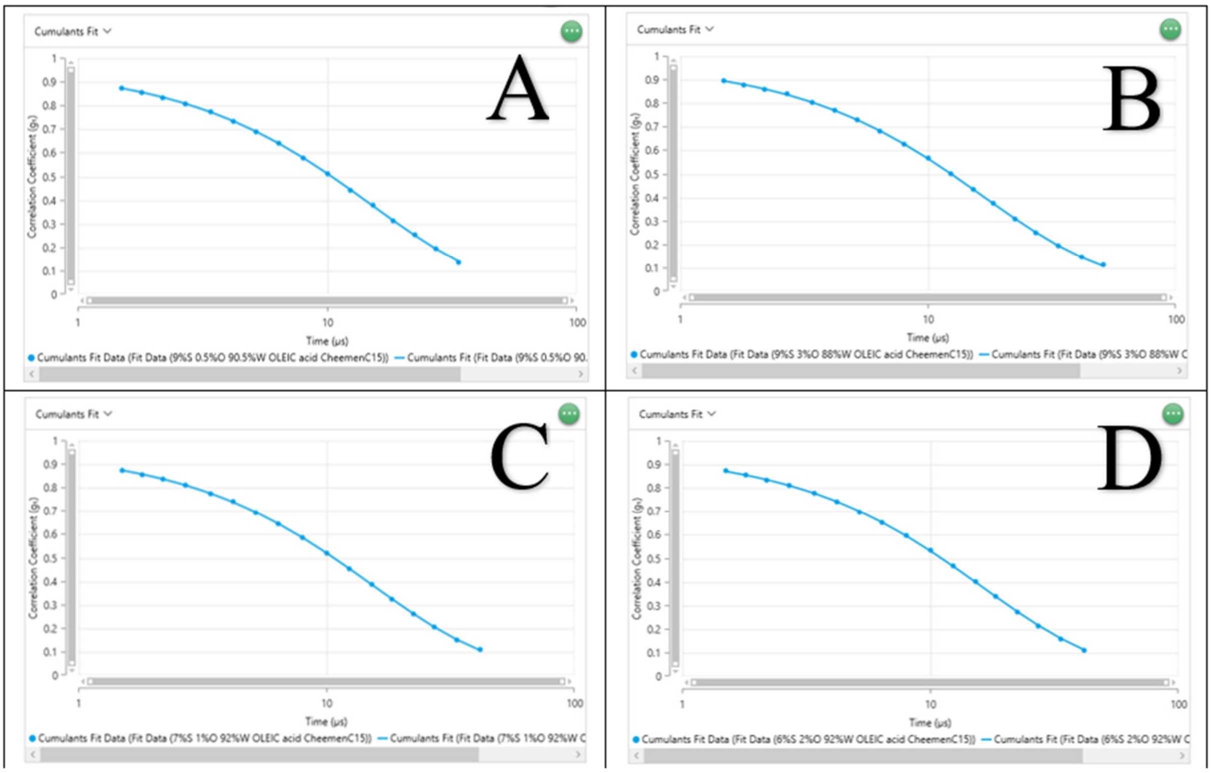

Figure S7. Cumulants fit for the studied microemulsion systems. Formulations: **A**—9% POE(15)cocoamine / 0.5% oleic acid / 90.5% water; **B**—9%

POE(15)cocoamine / 3% oleic acid / 88% water; **C**—7% POE(15)cocoamine / 1% oleic acid / 92% water; **D**—6% POE(15)cocoamine / 2% oleic acid / 92% water.

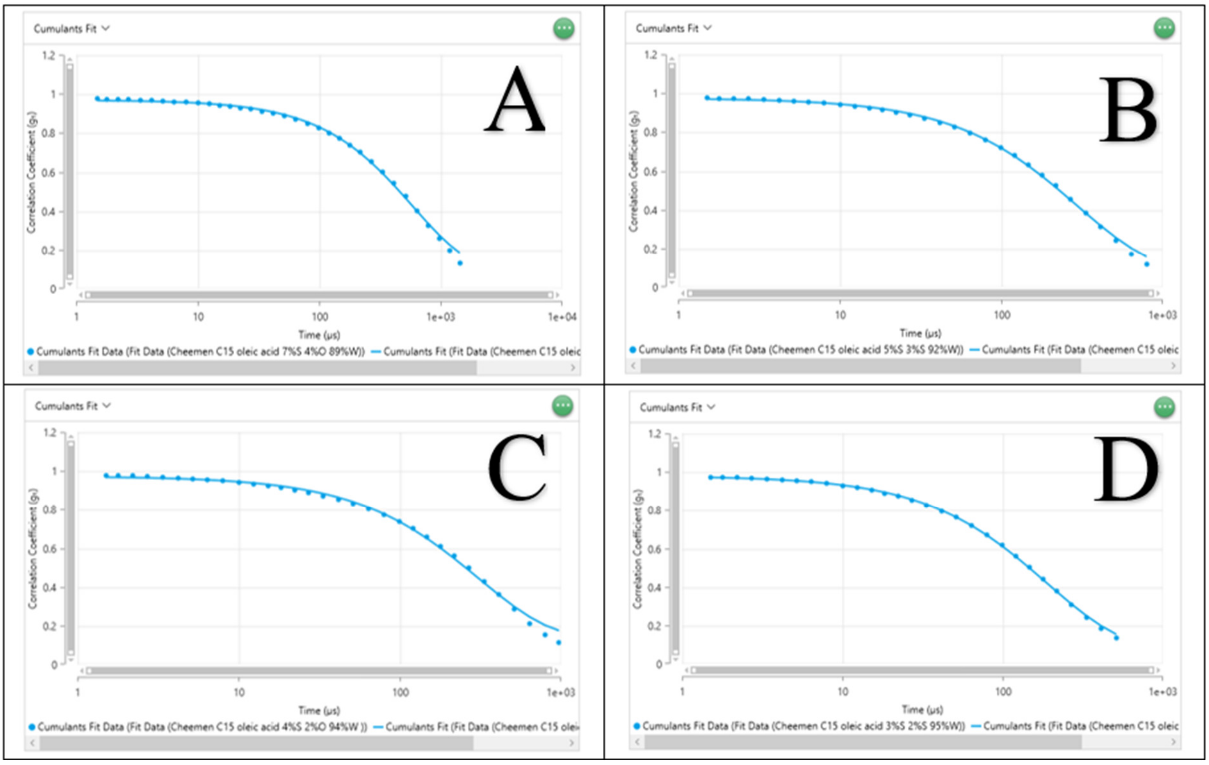

Figure S8. Cumulants fit for the studied nanoemulsion systems. Formulations: **A**—7% POE(15)cocoamine / 4% oleic acid / 89% water; **B**—5% POE(15)cocoamine / 3% oleic acid / 92% water; **C**—4% POE(15)cocoamine / 2% oleic acid / 94% water; **D**—3% POE(15)cocoamine / 2% oleic acid / 95% water.

The good agreement between measured and model-fitted data confirms the reliability of the obtained hydrodynamic diameters. Additionally, low residuals and consistent PDI values further support the analysis and confirm the reliability of the reported hydrodynamic diameters.

All measurements were carried out using the advanced Zetasizer Xplorer software, which by default applies the Stokes–Einstein equation for particle size analysis. Accurate parameter input during measurement setup is crucial, particularly regarding the solvent properties. In our case, water was selected as the dispersant as it constitutes the majority of our formulations. The Zetasizer Xplorer software includes pre-defined physical constants for water: a refractive index (n) of 1.33 and a dynamic viscosity (η) of 0.8872 mPa·s at 25°C. To ensure full compliance with the technical assumptions underlying the Stokes–Einstein model, it is also worth mentioning that all measurements were performed using the standard 173° backscattering detection configuration. According to Malvern Zetasizer pro manual, the use of backscattering minimizes multiple scattering and reduces the impact of dust or large contaminants,

thereby enhancing the accuracy and reliability of particle size determination. Combined with correct solvent parameters and rigorous sample preparation, it guarantees that the hydrodynamic diameter values reported are valid.

It should be noted that one of the fundamental assumptions for the application of the Stokes–Einstein equation in DLS analysis is that particles must exhibit translational Brownian motion in the dispersing medium. This condition ensures that diffusion behavior reflects only random thermal movement and is not influenced by external forces or convection [based on the information in the manual provided by Malvern Company]. In our study, this requirement was fulfilled by performing measurements in a freshly prepared cuvette, under precisely controlled temperature conditions, with an equilibration time of 120 seconds applied both before and between measurements to ensure thermal and hydrodynamic stability. Secondly, the particles are expected to be spherical and monodisperse. In our study, the samples under investigation meet the required physical assumptions as both microemulsions and nanoemulsions are, by their very nature, typically spherical. Emulsion systems are formed by minimizing interfacial tension, thereby promoting spherical morphology. This shape is thermodynamically favored as the system naturally tends toward energy minimization, and a sphere provides the maximum internal volume with the minimal surface area [53]. The low polydispersity index (PDI < 0.1) obtained from our measurements further supports the assumption of spherical and narrowly distributed nanoparticles.

Furthermore, all solvent parameters necessary for DLS modeling, specifically, viscosity and refractive index, were well-defined and entered with high accuracy. Deionized water, which constituted the continuous phase of our emulsions, was used as the dispersant. Its viscosity ($\eta = 0.8872$ mPa·s) and refractive index ($n = 1.33$ at 25 °C) are well-characterized and embedded in the instrument software's standard database, ensuring consistency and eliminating uncertainty in the model inputs. Lastly, the system did not contain polymeric coatings or additional substances that might alter the apparent hydrodynamic layer around particles, which could influence interparticle forces or introduce hydration shells of variable thickness—crucial for the accurate interpretation of DLS results using the Stokes–Einstein relation [54]. Based on these conditions, we can confirm that the hydrodynamic diameter and PDI values derived from our DLS measurements are analytically valid. We have included a series of consecutive datasets from the raw correlation $g_2(q, \tau)$ data to the size/radii distribution functions. Briefly, the hydrodynamic radii were calculated from $g_2(q, \tau)$ using Siegert

relation and CONTIN algorithm [55–58]. In our analysis, we used the software provided by the instrument vendor (Malvern).

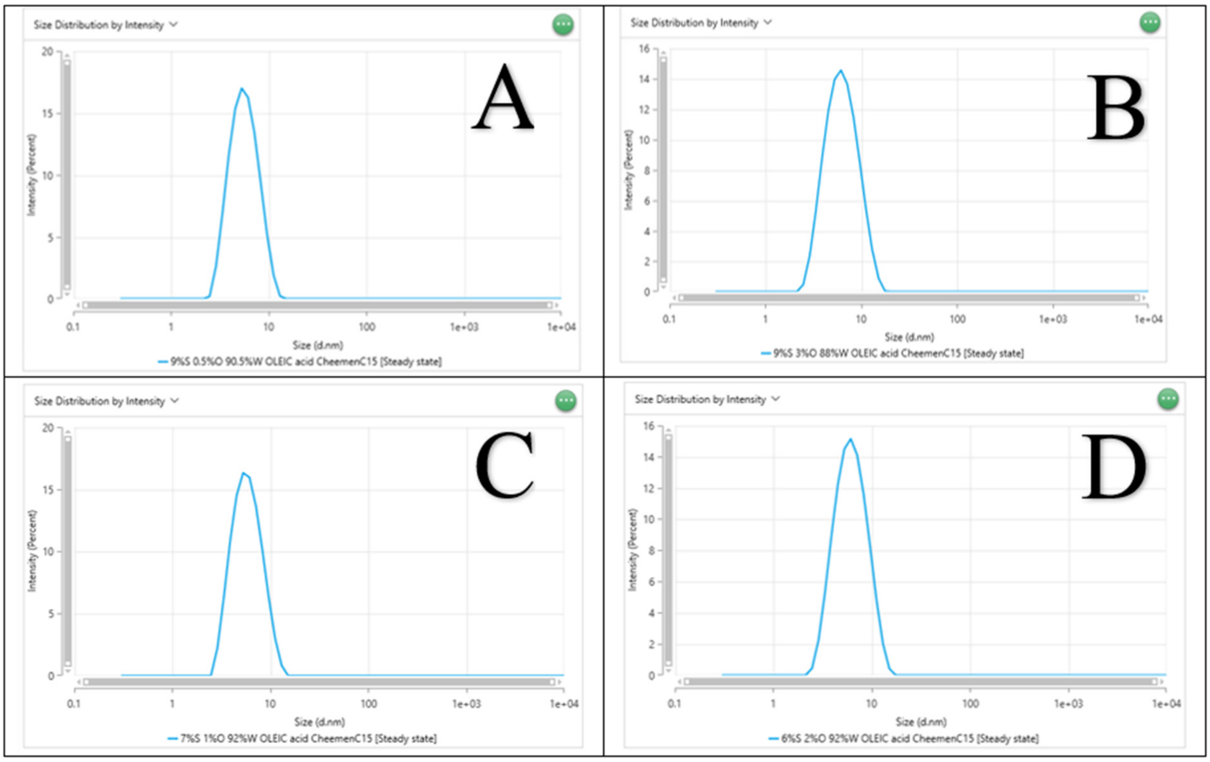

Figure S9. Obtained size distributions by intensity for the studied microemulsion systems.
Formulations: **A**—9% POE(15)cocoamine / 0.5% oleic acid / 90.5% water; **B**—9% POE(15)cocoamine / 3% oleic acid / 88% water; **C**—7% POE(15)cocoamine / 1% oleic acid / 92% water; **D**—6% POE(15)cocoamine / 2% oleic acid / 92% water.

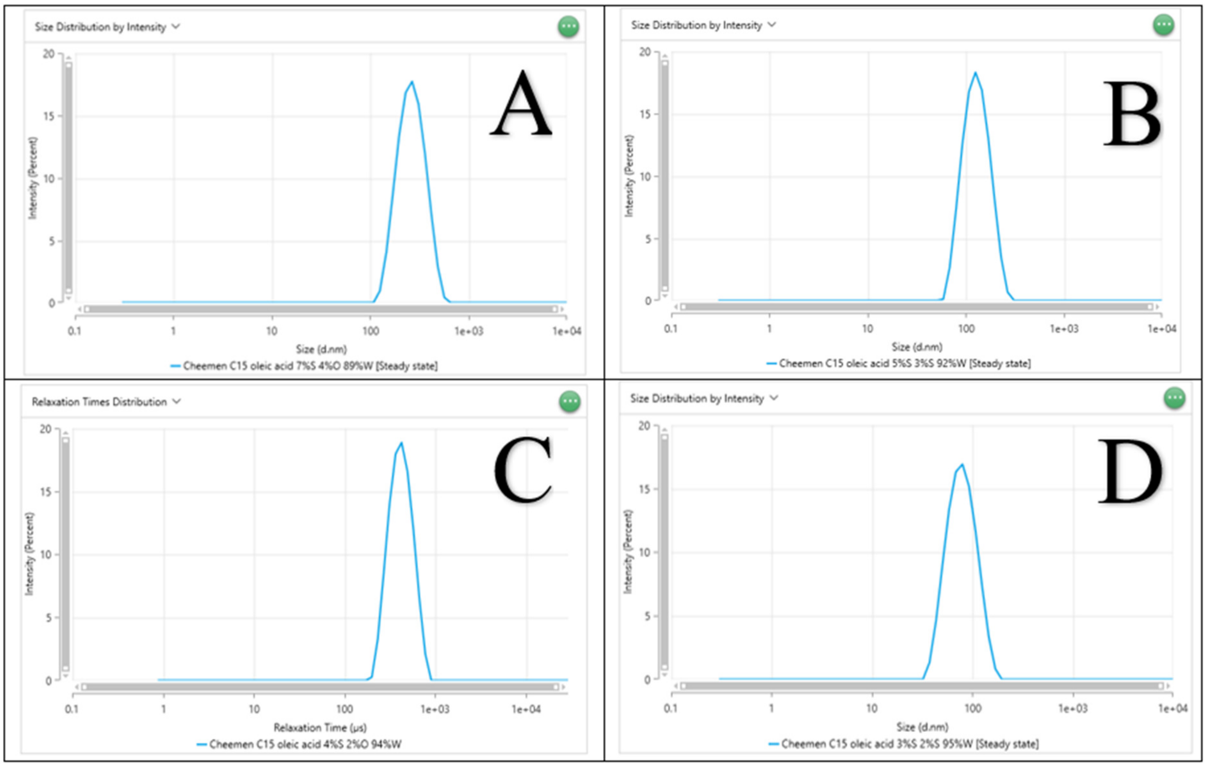

Figure S10. Obtained size distributions by intensity for the studied nanoemulsion systems.

Formulations: **A**—7% POE(15)cocoamine / 4% oleic acid / 89% water; **B**—5% POE(15)cocoamine / 3% oleic acid / 92% water; **C**—4% POE(15)cocoamine / 2% oleic acid / 94% water; **D**—3% POE(15)cocoamine / 2% oleic acid / 95% water.
